# A novel fluorescent probe, triphenylamine rhodamine-3-acetic acid (mRA) for the detection of Amyloid-β aggregates in Alzheimer's disease

**DOI:** 10.3389/fnins.2025.1653063

**Published:** 2025-08-21

**Authors:** Raja Chinnappan, Mateen A. Khan, Taj Mohammad, Sarah Mohammed Allwaibh, Shanmugam Easwaramoorthi, Ahmed Yaqinuddin, Sandhanasamy Devansan, Tanveer Ahmad Mir, Imtaiyaz Hassan

**Affiliations:** ^1^College of Medicine, Alfaisal University, Riyadh, Saudi Arabia; ^2^Tissue/Organ Bioengineering & BioMEMS Laboratory, Organ Transplant Centre of Excellence (TR & I-Dept), King Faisal Specialist Hospital and Research Centre, Riyadh, Saudi Arabia; ^3^Department of Life Sciences, College of Science and General Studies, Alfaisal University, Riyadh, Saudi Arabia; ^4^Center for Interdisciplinary Research in Basic Sciences, Jamia Millia Islamia, Jamia Nagar, New Delhi, India; ^5^Inorganic and Physical Chemistry Lab, CSIR-Central Leather Research Institute, Adyar, Chennai, India; ^6^Bioproducts Research Chair, Department of Zoology, College of Science, King Saud University, Riyadh, Saudi Arabia

**Keywords:** Alzheimer's disease, mRA, fluorescent sensing probe, binding, thermodynamics, molecular docking, conformational change

## Abstract

Amyloid-β (Aβ) is implicated in the pathophysiology of Alzheimer's disease (AD) and plays a significant role in neuronal degeneration. Aβ in solution is essential during the initial stages of developing lead compounds that influence Aβ fibrillation. The tendency of the Aβ peptide to misfold in solution is correlated with the etiology of AD. Therefore, the early detection of Aβ serves as a critical foundation for diagnostic testing and routine clinical assessment of AD. Herein, an aggregation-induced fluorescence probe, triphenylamine rhodamine-3-acetic acid (mRA), was used to detect Aβ aggregates. The fluorescence results showed a strong interaction between the fluorescence probe mRA and Aβ aggregates. mRA specifically binds with high affinity to Aβ aggregates, and the limit of detection (LOD) of Aβ aggregates was 0.12 μg/mL. Molecular docking studies showed that the mRA has significant binding affinity toward the Aβ peptide at the N/C-terminal region, with a binding energy of −6.5 kcal/mol. Furthermore, CD studies confirmed that the mRA binds to Aβ aggregates, and its binding induces significant structural alteration of the Aβ aggregates. Thermodynamic properties revealed that the binding of Aβ aggregates to mRA is a spontaneous process, driven by enthalpy and favored by entropy, which helps further our understanding of the interaction between mRA and Aβ aggregates at the molecular level. The negative ΔH suggests that hydrogen bonding is a dominant force for the mRA interaction with Aβ aggregates. This study provides a rationale for using mRA as a biosensor for the detection of Aβ aggregates in biological fluids, offering a potential tool for the early diagnosis and monitoring of amyloid progression in AD.

## 1 Introduction

**Scheme 1:**
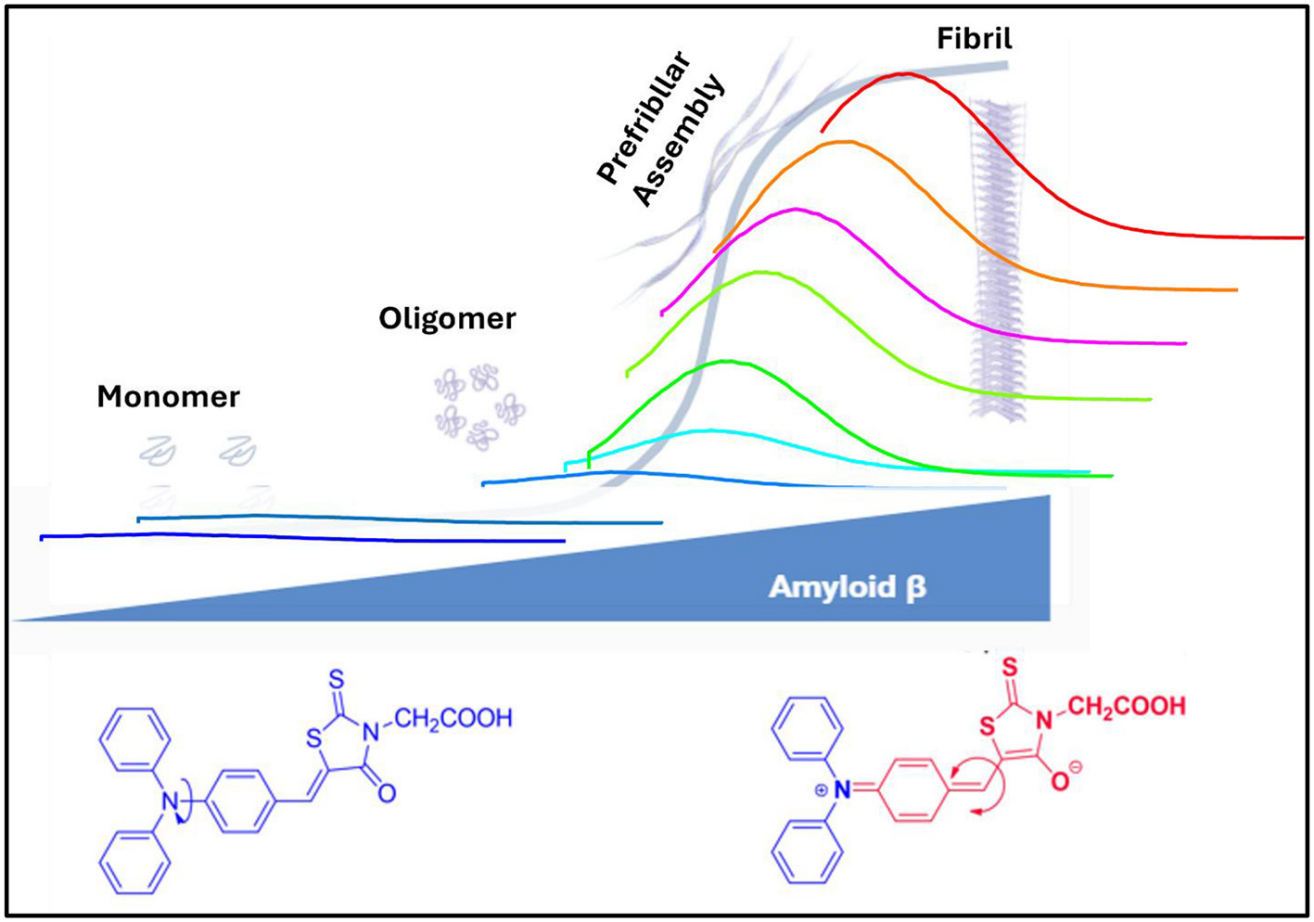
Schematic representation of monitoring the Aβ protein by fluorescence probe mRA.

Alzheimer's disease (AD) is a neurodegenerative condition marked by amyloid plaques and neurofibrillary tangles building up and progressive neuronal destruction (Scheltens et al., 2016; Hampel et al., 2021). In contrast to internal neurofibrillary tangles, which are composed of aggregated phosphorylated tau, external amyloid plaques have been found to consist mostly of aggregated Amyloid-β (Aβ) (Avila et al., 2004). The main component of senile plaques in AD brains is the longest and most hydrophobic. In a normal human brain, Aβ_1 − 40_ is predominant; however, in disease conditions, excess Aβ_1 − 42_ is found in cerebrospinal fluid (CSF) and predominantly accumulated as amyloid plaques. A soluble Aβ is derived from amyloid precursor protein (APP) (integral membrane protein) by the amyloidogenic pathway's successive cleavage of β-secretase and γ-secretase (Selkoe, 2001). However, depending on the physiological environment, Aβ can accumulate into soluble oligomers and protofibrillar intermediates, which subsequently aggregate into insoluble fibrils and amyloid plaques. The Aβ_1 − 42_ has severe neurotoxicity and possesses faster aggregation kinetics compared to Aβ_1 − 40_ (Kayed et al., 2009)_._ The reduction of soluble Aβ due to its oligomerization and aggregation may serve as an early biomarker for Alzheimer's disease (AD). Abnormal synthesis and accumulation of Aβ in blood plasma and cerebrospinal fluid (CSF) are key pathological events contributing to the progression of AD. The deposition of amyloid on neurons leads to neurodegeneration and cognitive decline, which are hallmarks of dementia and early indicators of Alzheimer's disease. Therefore, routine clinical analysis and personalized treatment strategies rely on the early and accurate detection of amyloid species in CSF and other biological fluids (Scheme 1). Among the various Amyloid-Beta isoforms, Aβ_1 − 42_ is the principal component involved in amyloid plaque formation and represents a promising biomarker for monitoring Alzheimer's disease progression. The detection of Aβ levels in human cerebrospinal fluid (CSF) has been widely validated as a reliable biomarker for the diagnosis of Alzheimer's disease, including at its earliest clinical stages (Qin et al., 2018).

Traditional imageology tests and clinical symptoms are the primary methods used to diagnose AD. However, because of its gradual onset, symptoms frequently develop later than the disease progresses (Nasica-Labouze et al., 2015). For the early diagnosis and treatment of AD, it is crucial to create non-invasive, quick, and trustworthy methods. Various techniques and attempts have been made for screening Aβ. The enzyme-linked immunosorbent assay (ELISA) is widely used to measure Aβ peptides (Barghorn et al., 2005). Recently, an electrochemical and fluorescence sensing methodology has been widely adopted for probing Aβ aggregation and its interaction with biomolecules (Qin et al., 2018; Sharma et al., 2022). Several nanomaterials were created to find biomarkers based on CSF (Qin et al., 2019). Raman dye-coated polyA aptamer-AuNPs and the surface-enhanced Raman scattering (SERS) technology have been used to produce a SERS biosensor platform for the detection of Aβ_1 − 42_. These techniques are cumbersome for point-of-care applications with limited resources. The fluorescence technique has numerous advantages over these other technologies, including quantitative detection, low-cost equipment, real-time analysis, remote detection capability, specificity, high sensitivity, and no sample preparation complexity. Moreover, fluorescent methods provide longer-term, real-time biointeraction monitoring and are non-invasive (Kubota and Hamachi, 2015).

Biosensors have been crucial in identifying Aβ aggregates and monomers in recent years. Aβ in CSF and blood samples has been measured using the current detection techniques, which are based on electrochemical, surface plasmon resonance (SPR), SERS, and colorimetric sensors. As a novel approach for the quantitative measurement of Aβ, fluorescent probe sensors have garnered increasing attention due to their high sensitivity, rapid response times, and suitability for Aβ detection and analysis (Ren et al., 2020; Fang et al., 2023). Moreover, fluorescent dyes are relatively easy to design, modify, and synthesize. A variety of turn-on and environment-sensitive probes have been developed. Several fluorescent probes have demonstrated enhanced selectivity and photostability, offering improved performance for detecting Aβ aggregates in complex biological environments (Zhang et al., 2021; Mallesh et al., 2022, 2023). Aggregation-induced emission (AIE) probes display minimal background signal and selectively light up upon binding to Aβ aggregates (Fang et al., 2023). Furthermore, Förster resonance energy transfer (FRET)-based biosensors have enabled real-time monitoring of Aβ aggregation (Trusova et al., 2022). These advances highlight the growing utility of fluorescence probe–based methods in both fundamental research and potential diagnostic applications for AD.

Recently, fluorescence turn-on probes have been employed for the detection of Aβ, with Thioflavin T (ThT) and Congo Red being among the most commonly used for quantitative detection of Aβ_1−_4_2_ (Howie and Brewer, 2009). ThT, in particular, is often considered the gold standard to detect aggregated Aβ_1 − 40_ and Aβ_1−_4_2_ and other fibrillary aggregates. However, its low binding affinity, limited selectivity, and poor specificity have driven the development of more effective fluorescent probes. In response to these limitations, a novel fluorescent molecule, Triphenylamine Rhodamine-3-Acetic Acid (mRA), has been designed and synthesized for the selective detection of Aβ aggregates.

In this study, we developed a turn-on fluorescence probe, mRA, for the selective detection of trace levels of Aβ_1 − 42_ aggregates in bodily fluids based on changes in the fluorescence-sensitive local microenvironment within the Aβ_1 − 42_ aggregates mRA complex fluorescence. mRA has weak fluorescence in PBS buffer; however, a strong emission signal is observed upon binding to Aβ aggregates. The well-designed fluorescent probe mRA selectively and sensitively recognizes Aβ_1 − 42_, exhibiting high binding affinity and excellent thermostability in the resulting complex. This study provides a relevant sensing approach for the growing number of dyes binding small molecules to assist in the early detection of Aβ aggregates in AD.

## 2 Materials and methods

### 2.1 Materials

Triphenylamine rhodamine-3-acetic (mRA) dye was synthesized as described previously (Thamaraiselvi et al., 2019). The Aβ protein was purchased from OriGene (Rockville, MD, USA). The concentration of protein was determined following the standard Bradford method (Bradford, 1976). All buffers were made up in Milli-Q water with a resistivity of 18.2 MΩ.cm. All the other chemicals used in this study were of molecular biological grade.

### 2.2 Steady state fluorescence measurement

Fluorescence spectroscopy was used to determine the strength of binding affinity of the fluorescence probe, mRA, with Aβ protein. The fluorescence probe mRA bound to Aβ was observed in the emission range of 510–750 nm by exciting the complex at 475 nm. The excitation and emission slits were set at 5 and 10 nm, respectively, with a cuvette path length of 1 cm. In the fluorescence experiments, Aβ concentrations were varied from 0 to 300 μg/mL, while the constant concentration of mRA was maintained at 2.5 μM. All spectral measurements were made after incubating mRA-Aβ_1 − 42_ in PBS buffer (pH 7.4) for 15 min to keep the necessary temperature constant. A linear relationship was observed between Aβ_1 − 42_ and mRA, as demonstrated by a linear plot of Aβ_1 − 42_ concentration vs. mRA fluorescence intensity. The minimum detectable amount of Aβ_1 − 42_, or the limit of detection (LOD), was calculated using the standard formula:


$$\begin{array}{l} \text{LOD}=\frac{3.3\sigma }{S} \end{array}$$

Where σ is the standard deviation of the blank signal (without BSA); S is the slope of the calibration plot.

All sample binding investigations were conducted at 25°C under dim light to protect from photo-oxidation of the fluorescent probe. A thermocouple device was used to control the cuvette holding sample temperature (Δ*T* ± 0.1°C). The titration of mRA with increasing Aβ concentrations was carried out in a discontinuous manner. To a fixed concentration of mRA (2.5 μM), increasing amounts of Aβ were added from 0 to 300 μg/mL. After adding Aβ, the changes in mRA fluorescence intensity data were analyzed by fitting them using the following equation:


(1) $$\begin{array}{l} \Delta F=\;\left(F_{0}-F_{\text{f}}\right)/F_{0} \end{array}$$

Where Δ*F* is the change in fluorescence enhancement seen in any sample; the fluorescence intensity of mRA alone (control) is denoted by *F*_0_; *F*_*f*_ denotes the fluorescence enhancement after adding Aβ. To account for the background signal, the fluorescence of Aβ_1 − 42_ alone in PBS buffer was subtracted from the measured fluorescence intensities of the Aβ_1 − 42_/mRA complex. The resulting corrected fluorescence values were then used to calculate the binding affinity of the complex. The equilibrium binding affinity (*K*_*a*_ = 1/*K*_*d*_) was determined using the normalized fluorescence values (ΔF/Δ*F*_max_), where Δ*F*_max_ represents the maximum fluorescence change corresponding to complete saturation of mRA by Aβ_1 − 42_. To determine Δ*F*_max_, a 1/ΔF vs. 1/[Aβ] plot was extrapolated to the ordinate for the intercept (Khan et al., 2023). The equilibrium measurement was conducted using three independent titration trials. *K*_d_ values were acquired by KaleidaGraph using non-linear regression analysis.

We further investigated the temperature-dependent Aβ binding to mRA. This assay was carried out at four distinct temperatures (288, 293, 298, and 310 K) under the same standard experimental conditions as the fluorescence titration described above. The experimental temperature was maintained for each sample using a temperature controller.

### 2.3 Thermodynamic measurements

To better understand the mechanism of interaction for the binding forces involved in the binding of mRA and Aβ_1 − 42_ aggregates, thermodynamic measurements were performed. Temperature-dependent binding constant for the mRA-Aβ_1 − 42_ aggregates complex formation was monitored by the fluorescence measurements as described previously (Khan et al., 2017). Change in enthalpy (ΔH) and entropy (ΔS) between Aβ_1 − 42_ aggregates-mRA were determined by Van't Hoff equation. Equation (3) was used to calculate the change in Gibbs free energy involved in an Aβ_1 − 42_/mRA complex formation.


(2) $$\begin{array}{l} ln\;K_{a\;}=\;-\frac{\Delta H}{R\;T\;}+\;\frac{\Delta S}{R} \end{array}$$

(3) $$\begin{array}{l} \Delta G=\;-\;R\;T\;ln\;K_{a}=\;\Delta \text{H }-\text{ T}\Delta \text{S} \end{array}$$

Where *K*_a_ is the binding constant; Δ*H* and Δ*S* are the enthalpy and entropy change; Δ*G* is the Gibbs free energy; *T* is the absolute temperature; *R* is the universal gas constant (1.987 Cal mol^−1^ K^−1^). The intercept and slope of a plot of ln *K*_a_ vs. the inverse of temperature yielded –Δ*H*/*R* and Δ*S*/*R*.

### 2.4 Circular dichroism spectroscopy

Far-UV circular dichroism (CD) spectra of native Aβ protein and Aβ_1 − 42_/mRA were observed with a spectropolarimeter. We employed a thermo-controller with a circulating water bath to keep the temperature at 298 K under a continuous flow of nitrogen gas with a slit width of 2 nm. As directed by the manufacturer, D-10-camphorsulphonic acid was used to calibrate the instrument. Changes in ellipticity were obtained at a fixed Aβ_1 − 42_ concentration (1 μM) and in the presence of varying amounts of mRA (0–10 μM). After 15 min of incubation, spectra were recorded for each sample.

Each CD spectrum was acquired using a 0.1 cm quartz cell in the wavelength range of 190–260 nm. The instrument response time was one second with a recording rate of 100 nm/min. Each spectrum was obtained by averaging five scans before the final spectral collection. Each collected spectrum was corrected by subtracting the corresponding contributions from the buffer alone and, when applicable, the Aβ_1 − 42_/mRA complex in buffer. The obtained spectra were confirmed by eliminating a buffer background scan from the initial protein spectrum. Every spectrum having a bandwidth of 60 was subjected to the SG filter. Each spectrum data set was displayed as mean residual ellipticity vs. wavelength (nm) as a function of (deg.cm^2^.dmol^−1^). The change in the intensity of the free Aβ_1 − 42_ ellipticity upon addition of mRA suggests a conformational change in the Aβ_1 − 42_ sample in the presence of different mRA concentrations, which was calculated using the CDNN tool.

### 2.5 Molecular docking analysis

Molecular docking studies were conducted to investigate the binding of mRA with Aβ_42_ residue peptide (APPβ) using Insta Dock v1.2 (Mohammad et al., 2021). The solution structure of the Aβ peptide (1-42) was retrieved from the RCSB Protein Data Bank (PDB ID: 1Z0Q) (Bank, 1971). The structures were prepared by adding hydrogen atoms, assigning charges, and defining appropriate atom types. A blind docking approach, covering the entire APPβ proteins, was adopted to identify binding sites. The 2D molecular structure of mRA was drawn using ChemBioDraw Ultra 14.0 and converted to a 3D structure, followed by geometry optimization using the MMFF94 force field before docking (Supplementary Figure S1). Flexible docking simulations employed grid dimensions of 58 Å × 51 Å × 30 Å for Aβ peptide, centered at coordinates (−5.364, −0.161, −5.029). The grid spacing was set to 1.00 Å, and the exhaustiveness parameter was adjusted to 8 for thorough conformational sampling. Docking simulations were executed with default parameters, ranking the resulting poses based on binding affinity and interaction energy. Key interactions between mRA and amino acid residues in the binding sites were analyzed. The top-ranked conformations were processed using the InstaDock Splitter program, and detailed binding modes were visualized using PyMOL (DeLano, 2002) and Discovery Studio Visualizer (Visualizer, 2005).

## 3 Results and discussion

### 3.1 Interaction of mRA probe with Aβ protein

**Figure 1:**
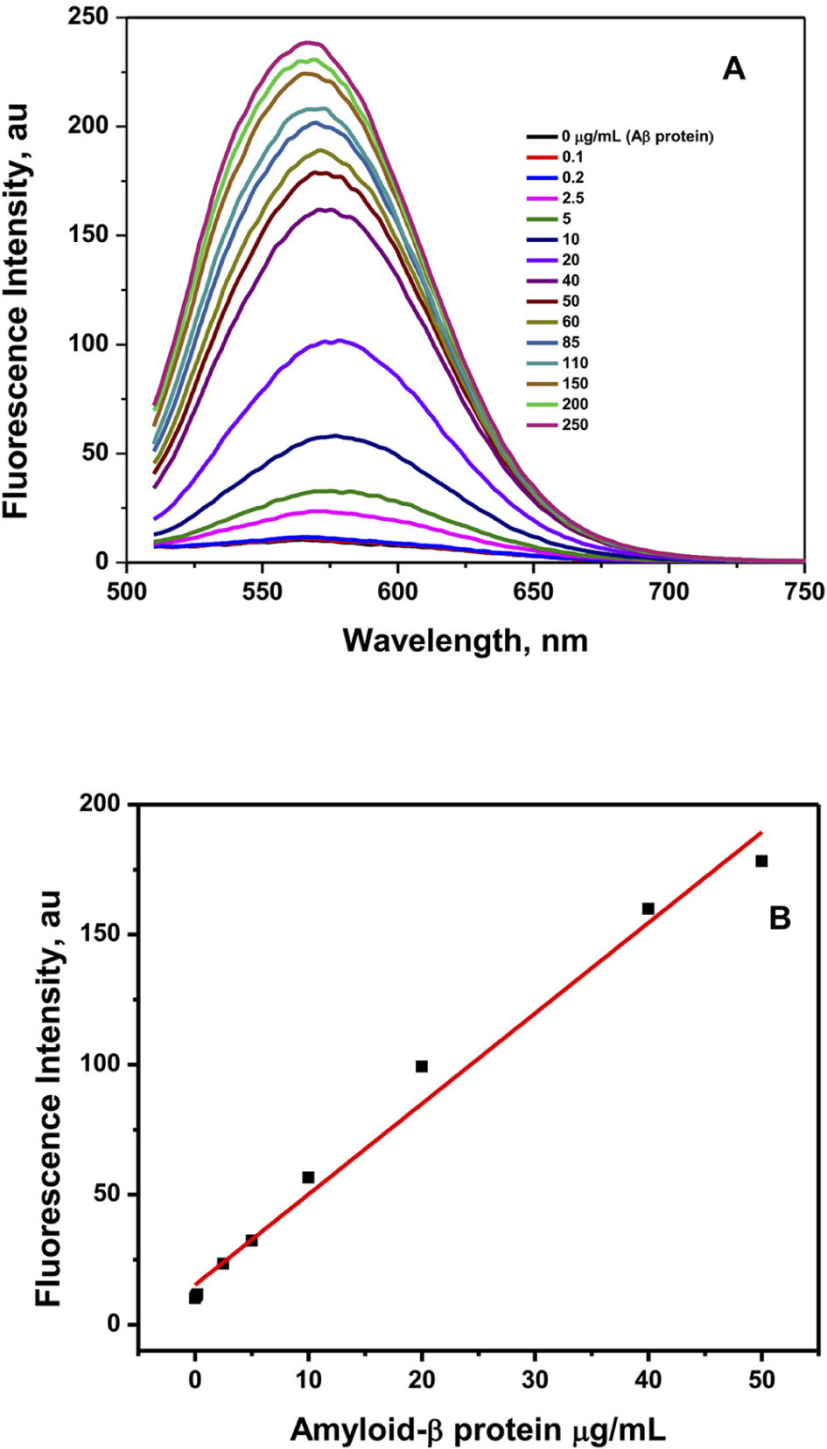
Fluorescence titration spectra of mRA probe binding to Aβ. **(A)** Fluorescence enhancement of mRA probe (2.5 μM) with increasing concentrations of Aβ (0–10 μM) at 298 K. The spectra of mRA binding to Aβ were measured by exciting the samples at 475 nm, and emission between 500 and 750 nm. **(B)** Fluorescence intensity measurements as a function of Aβ concentration.

Binding interactions between the mRA probe and Aβ were examined through fluorescence enhancement titration, molecular docking studies, CD spectroscopy, and specificity and cross-reactivity analyses. It is known that Aβ_1 − 42_ exhibits different forms of aggregation in increasing concentrations, and the aggregated forms interact with the probe and influence their fluorescence behavior (Scheme 1). Excitation of mRA/Aβ_1 − 42_ aggregate complex at 475 nm produced a fluorescence in the range of 500–750 nm with an emission peak at 575 nm. Figure 1A shows fluorescence emission spectra of mRA in the presence of increasing concentrations of Aβ_1 − 42_. A fixed concentration of mRA (2.5 μM) was titrated with varying amounts of Aβ_1 − 42_ in the range of 0–300 μg/mL. A concentration-dependent increase in fluorescence intensity was observed with Aβ, suggesting an interaction with mRA that alters its fluorescence characteristics. As reported previously, the critical aggregation concentration (CAC) of Aβ_1 − 42_ is 90 nM (Novo et al., 2018). The observed enhancement in fluorescence intensity suggests that monomeric Aβ_1 − 42_ undergoes progressive aggregation into various forms, such as oligomers, prefibrillar assemblies, protofilaments, and mature fibrils as its concentration increases. The maximum fluorescence intensity observed at 300 μg/mL of Aβ_1 − 42_ indicates that the mRA probe exhibits a stronger interaction with mature fibrils compared to other aggregate forms. Figure 1B depicts a linear relationship established between the concentration of Aβ_1 − 42_ and the fluorescence response of mRA. The limit of detection (LOD), calculated from the linear plot, was determined to be 0.12 μg/mL.

**Figure 2:**
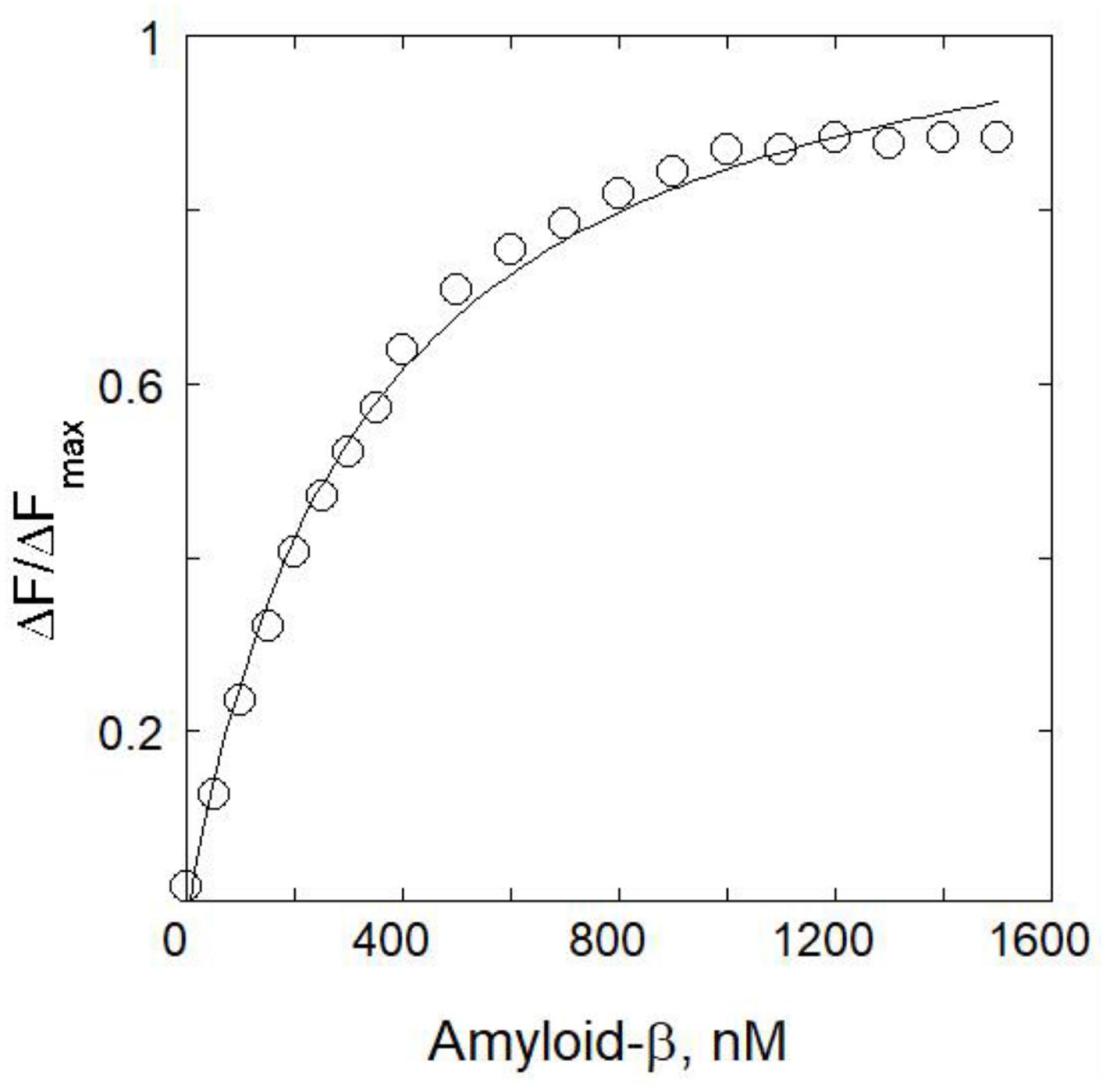
The mRA probe strongly interacts with the Aβ-peptide. Direct fluorescence titration of Aβ at 25°C was used to measure the affinity of mRA for Aβ. Various amounts of Aβ were incubated with constant mRA (1 μM) to prepare the samples.

By directly titrating Aβ with limiting mRA concentration, the interaction between Aβ and mRA was observed in equilibrium conditions. Figure 2 shows the binding of mRA with the increasing amounts of Aβ_1 − 42_. The addition of Aβ_1 − 42_ protein significantly enhanced the fluorescence intensity of the mRA probe. Fluorescence change revealed the protein and ligand binding, which promotes a change in orientation of the protein molecule induced by Aβ_1 − 42_ binding. Fluorescence enhancement of the mRA was observed when Aβ concentration increased. The peak for the native mRA sensor was located at 575 nm. On the other hand, mRA fluorescence increased as the quantity of Aβ_1 − 42_ increased, indicating the formation of an mRA/Aβ_1 − 42_ aggregate complex. The mRA sensor binds to Aβ_1 − 42_ with a significant binding affinity. The binding affinity for the mRA interaction to Aβ_1 − 42_ was 3.0 × 10^6^ M^−1^ at 298 K. The fluorescence data were analyzed by fitting them into binding plots as described in the method section.

In the early stages, Congo red, a deep crimson dye, was used for the detection of Aβ_1 − 42_. However, due to their poor performance and practical challenges, these dyes are being replaced by other efficient probes. Vassar and Culling demonstrated the diagnosis of amyloid fibrils by an amyloid-specific fluorescence probe called Thioflavin-T (ThT) by fluorescent microscopy (Puchtler et al., 1959). A drastic enhancement in the fluorescence upon binding to amyloid fibrils is explained by the selective immobilization of a specific group of the ThT molecule, and it behaves as a “molecular rotor.” Similar characteristic behavior of mRA was observed in the previous studies (Thamaraiselvi et al., 2019; Chinnappan et al., 2025). In the solution, the single bond between the nitrogen and the phenyl group attached to the acceptor moiety was rotating freely and rapidly, which leads to quenching the excited state and reducing the fluorescence emission. In contrast, the excited state mRA in the mRA/Aβ_1 − 42_ aggregate complex is unaffected by the quencher; the free rotational immobilization of the quencher in the complex does not influence the excited state of mRA, resulting in high fluorescence emission. As the concentration of Aβ increases, it forms strong amyloid fibrils that sterically lock the mRA probe (Figure 1). Therefore, the fluorescence emission increases proportionally to Aβ concentration.

**Figure 3:**
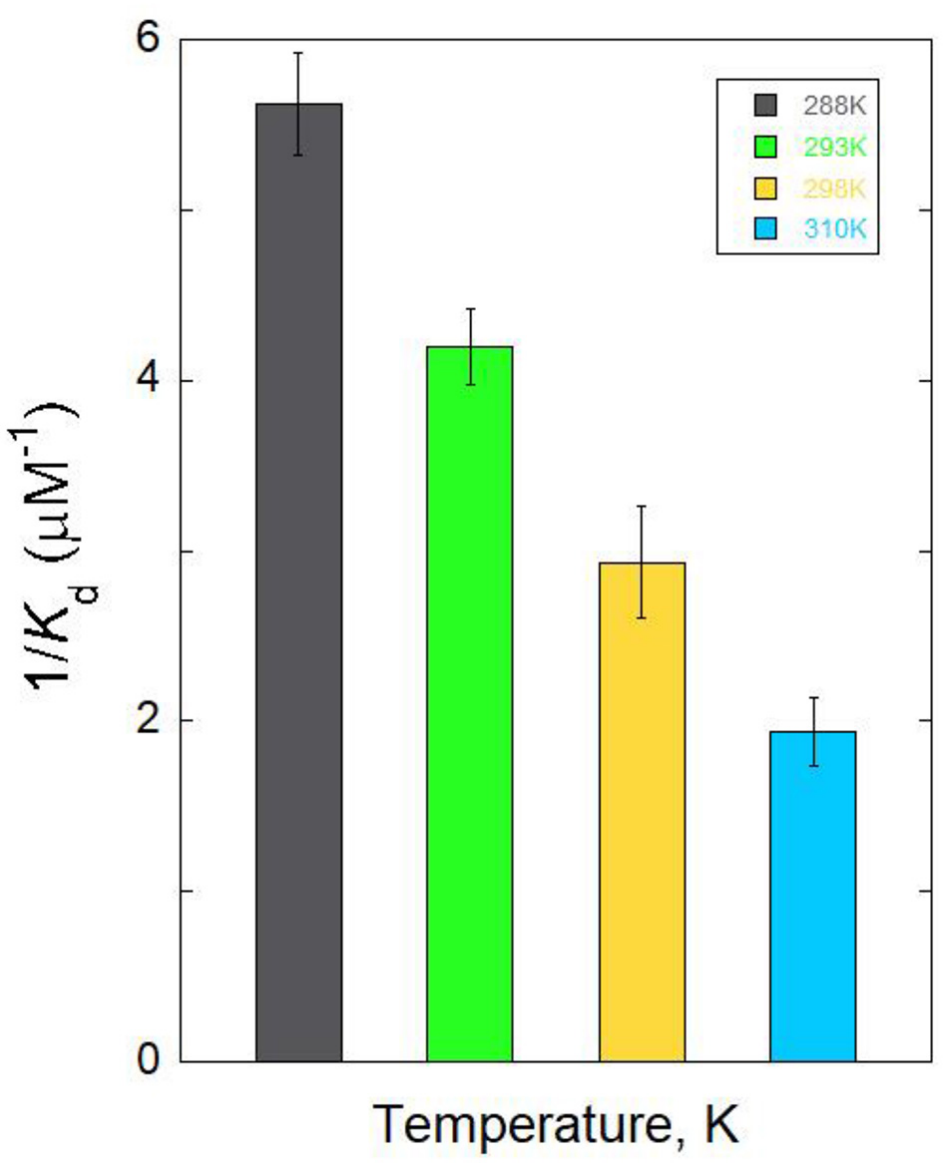
Temperature-dependent binding affinities of mRA/Aβ complex. Error bars represent the standard deviations calculated from three measurements.

Furthermore, using an intrinsic fluorescence assay at different temperatures (288, 293, 298, and 310 K), the binding characteristics of the mRA/Aβ_1 − 42_ aggregate interaction were investigated. The temperature-dependent binding affinity was used to evaluate the details of the mRA/Aβ_1 − 42_ aggregate's effective mechanism of interaction. Addition of Aβ_1 − 42_ to mRA at variable temperatures showed significant enhancement of mRA fluorescence, revealing that mRA binds to Aβ_1 − 42_ at different temperatures. Temperature-dependent binding parameters can offer insight into the operative mode of mRA/Aβ_1 − 42_ aggregate interaction. Figure 3 shows a summary of the binding affinity plot for mRA/Aβ binding at varying temperatures (Table 1). An increase in temperature led to a corresponding increase in the dissociation constant *K*_*d*_, indicating that mRA and Aβ form a dynamic, reversible complex. Specifically, the *K*_*d*_ increased from 178 ± 8.7 nM at 288 K to 515 ± 18 nM at 310 K, suggesting that the mRA/Aβ_1 − 42_ aggregate complex is more stable at lower temperatures. This temperature-dependent behavior highlights the thermally labile nature of the interaction. The *K*_d_ value of the mRA/Aβ_1 − 42_ aggregate binding at 310 K was greater than that at 288 K, according to fluorescence data analysis (Table 1). It was evident that the *K*_d_ increased with temperature, indicating a dynamic mode of interaction between mRA and Aβ_1 − 42_ aggregates.

**Table 1:** Dissociation constants (*K*_d_) for the binding of mRA probe with Aβ protein.

**Complex**			***K_*d*_* (nM)**	
	288 K	293 K	298 K	310 K
Aβ-mRA	178 ± 8.7	239.7 ± 11	341 ± 17	515 ± 18

### 3.2 Thermodynamics of mRA probe and Aβ binding

**Figure 4:**
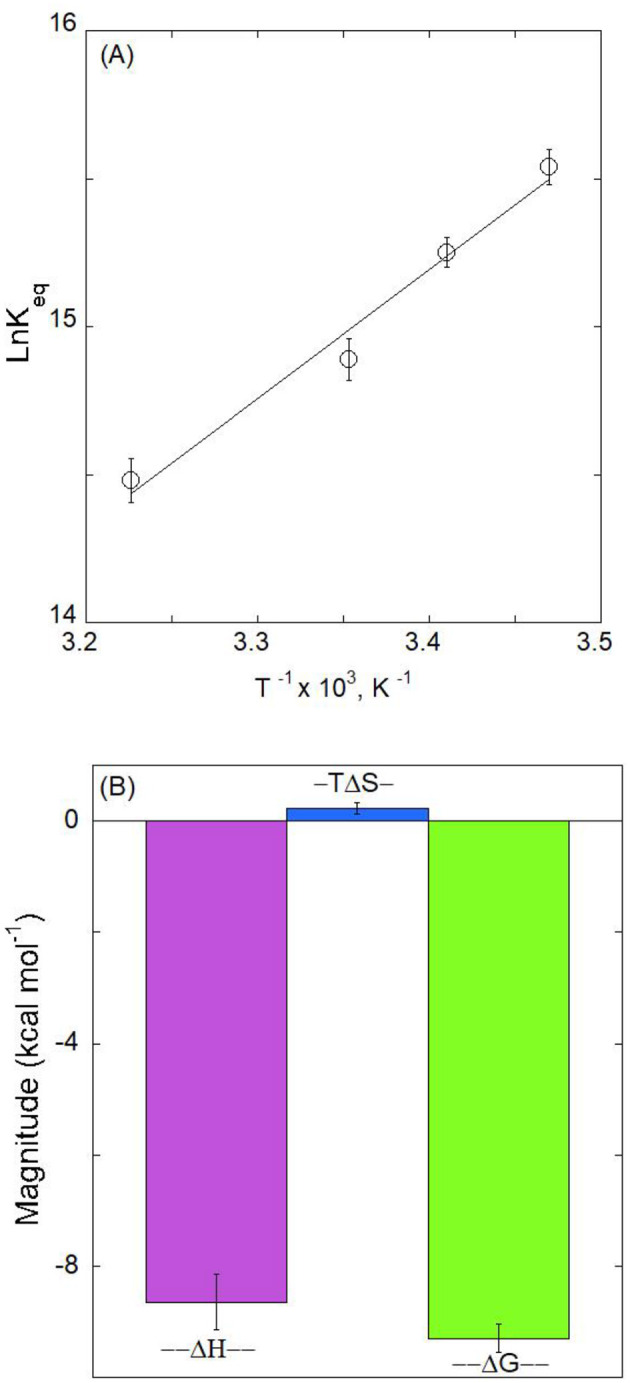
**(A)** Van't Hoff plot for binding mRA probe with Aβ. The slope and intercept of the plot ln*K*_avs._ 1/T were used to determine the ΔH and ΔS. The thermodynamic parameters for the binding of mRA to Aβ are shown in **(B)**. In the bar plot, ΔG, ΔH, and the entropy contribution to the free energy (–TΔS) of the binding of mRA to Aβ are shown. Error bars represent the standard deviation, mean ± SD, from the average value of the data from three measurements.

Thermodynamic features o the complex formation were investigated to gain a better understanding of the mechanism underlying an mRA/Aβ_1 − 42_ interaction. The stability of the mRA/Aβ_1 − 42_ β combination was evaluated utilizing thermodynamic parameters. By calculating binding affinity at various temperatures, the thermodynamics of the mRA/Aβ_1 − 42_ interaction aids in our understanding of the types of forces that lead to complex formation. Figure 4A shows Van't Hoff graph with T_−1_ on the x-axis and lnKa on the y-axis for the binding of mRA with Aβ_1 − 42_. Using the calculated K_*a*_ values in Table 1, the experimental temperature T, and the gas constant R, linear analysis of the Van't Hoff graph yielded results for enthalpy and entropy change. The slope provides the value of enthalpy change, and the intercept gives the value of △S. A small positive entropy (△S = 0.8 ± 0.04 cal mol^−1^ K^−1^) and a substantial negative enthalpy (△H = 8.7 ± 0.5 kcal mol-1) indicated that the interactions are enthalpically driven and favored by entropy (Khan et al., 2017). Additionally, △H negative (< 0) and T△S positive (>0) revealed that hydrogen bonding and van der Waals forces are the dominant forces involved in stabilizing mRA/Aβ_1 − 42_ aggregate complex formation.

The Δ*G* is a thermodynamic indicator of a binding reaction's spontaneity. A negative Δ*G* value indicates that the mRA/Aβ_1 − 42_ aggregate complex forms spontaneously under normal conditions. Figure 4 shows the thermodynamic characteristics of mRA binding to Aβ. The value of ΔG at 25°C was estimated using the variables from Table 2 and Equation 2. For the mRA/Aβ_1 − 42_ aggregate complex, the calculated value of ΔG was −9.3 ± 0.6 kcal mol^−1^. Additionally, molecular docking offers a thorough examination of the residues and forces that are essential to mRA/Aβ_1 − 42_ aggregate interactions.

**Table 2:** Thermodynamics for the interaction of the mRA probe and Aβ protein.

**Complex**	**Δ*G* (kcal mol^−1^)**	**Δ*H* (kcal mol^−1^)**	**Δ*S* (cal mol^−1^ K^−1^)**	**TΔ*S* (cal mol^−1^)**
Aβ-mRA	−9.3 ± 0.6	−8.7 ± 0.5	0.8 ± 0.04	229.5 ± 13.6

The negative ΔG value thermodynamically favors the mRA interaction with Aβ_1 − 42_. Both entropically (7.5%) and enthalpically (93%), the interaction between mRA and Aβ_1 − 42_ is advantageous, with an 85% higher enthalpic contribution to ΔG at 25°C. The involvement of the protein-ligand bonding forces was dependent on the sign and magnitude of the thermodynamic parameters (Ross and Subramanian, 1981; Khan, 2022). Additionally, a significant negative ΔH suggests that hydrogen bonding and van der Waals contact are the main driving forces that cause the formation of the mRA/Aβ_1 − 42_ aggregate complex (Ross and Subramanian, 1981; Tayyab et al., 2019). However, mRA/Aβ_1 − 42_ aggregate complexes showed a low *T*Δ*S* value, revealing the absence of hydrophobic forces.

### 3.3 mRA probe-induced conformational changes in Aβ protein

**Figure 5:**
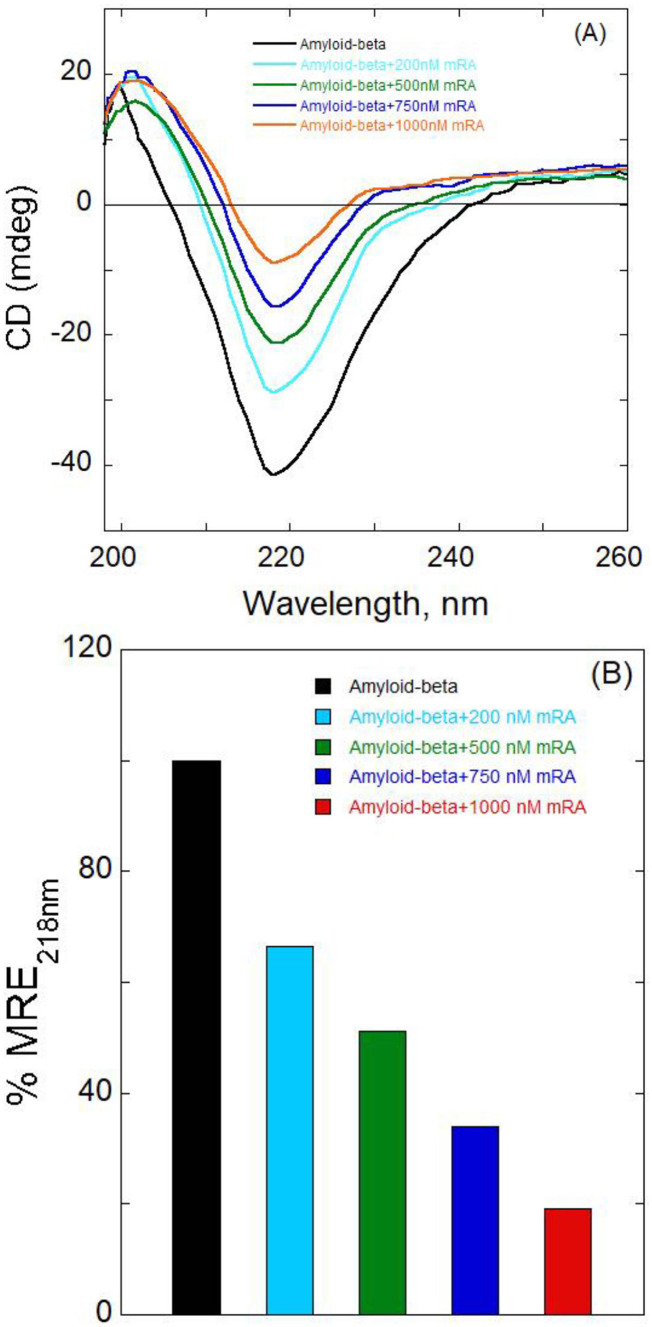
Far-UV circular dichroism spectra of Aβ at different Aβ to mRA probe molar ratios. The Aβ protein's ellipticity is considerably altered by adding mRA. (A) The far-UV CD spectra for Aβ (1 μM) in the absence or the presence of different amounts of mRA probe (0–10 μM). **(B)** Displays a bar plot that compares the percent mean residual ellipticity, MRE_218nm_ value of the native protein Aβ, and Aβ in complex with mRA probe.

At the secondary structural level of the protein, CD offers vital information on protein conformational properties. Using CD spectroscopy, one can examine structural alterations in proteins that result from interactions with ligands (Kelly and Price, 2000). Ligand binding often induces structural changes in proteins, making it one of the most important mechanisms for studying protein conformational dynamics (Bannister and Bannister, 1974). Protein conformation changes at the secondary structure level have been investigated with the help of the far-UV area CD measurements (Rodger et al., 2005; Khrapunov, 2009). CD spectra studies were conducted to assess alpha-helix, beta-sheet, and random coil. The effect of mRA on Aβ protein structural alteration was observed. Changes in the intensity of the spectral peak of proteins reflect secondary structural changes. Initially, the circular dichroism (CD) spectrum of native Aβ_1 − 42_ was examined to establish its baseline secondary structure. Subsequently, structural changes in Aβ_1 − 42_ upon binding to mRA were investigated to assess the conformational impact of the interaction. Figure 5A shows the graph of Aβ (1 μM) alone with a clear peak at around 218 nm, which is characteristic of a β-sheet conformation due to aggregation. Therefore, it is evident from this peak that β-sheets are the dominant structure of the Aβ_1 − 42_ protein. Addition of mRA at varying concentrations (0–10 μM) resulted in an intensity change, indicating binding of mRA to Aβ_1 − 42_ aggregates and altering the β-sheet structure of the amyloid protein aggregates.

This upward shift in the Aβ protein intensity brought on by the addition of mRA suggests that secondary structural modification is induced through mRA binding. The structural difference between free Aβ_1 − 42_ and Aβ_1 − 42_ bound to mRA is clear from the ellipticity change.

The insertion of mRA significantly affects the Aβ_1 − 42_ structure. After binding to the mRA, the intensity of Aβ_1 − 42_ protein changed, indicating a conformational alteration. Without a noticeable change in the shift of the Aβ_1 − 42_ peak, the change in band intensity indicates that mRA binding causes secondary structural changes. When mRA is added, Aβ_1 − 42_ ellipticity increases, suggesting the alteration of secondary structure in the protein.

Figure 5B displays the MRE_218_ change (%) of free Aβ_1 − 42_ and Aβ_1 − 42_ /mRA complex. Data revealed the influence of mRA on the change in ellipticity at 218 nm of Aβ_1 − 42_ binding to mRA. These findings showed that in the presence of mRA biosensor, the Aβ_1 − 42_ protein displayed structural modification. According to these findings, the Aβ_1 − 42_/mRA complex secondary structure composition changed significantly. Ellipticity values were measured as described elsewhere (Chen et al., 1972; Khan et al., 2002; Rabbani et al., 2014). After mRA treatment at 1,000 nM, the Aβ_1 − 42_ protein's secondary structure content dropped by 81%. We observed a decrease in the protein's helicity values upon the addition of mRA. These secondary structure changes demonstrate how the amyloid protein undergoes certain structural changes as a result of mRA binding. Given this notable structural change, it's plausible that mRA interacts with Amyloid-Beta via a beta sheet and that this conformational shift serves a detection purpose. Since mRA also changes the structure of the Aβ_1 − 42_/mRA complex, as observed in the CD spectra, it plays an important role in the helical loss of Aβ structure. Through the formation of hydrogen bonds, the secondary structure may influence both the stiff and flexible structure of proteins (Masuda et al., 2010). These studies revealed that the Aβ protein is β-sheet-rich.

Although the secondary structures of the protein were significantly changed, CD analysis reveals that the majority of Aβ could maintain structural integrity at or below 1,000 nM mRA. Thermodynamic parameters show the changes in free energy and enthalpy, which demonstrate that the complex's structure was altered by differences in the forces interacting between mRA and Aβ_1 − 42_. The results of thermodynamic and CD experiments are consistent with the fact that Aβ maintained its folded structure despite secondary structural changes. These findings were consistent with the hypothesis that mRA led to complex formation and structural changes in the Aβ_1 − 42_ protein, which were most likely brought on by the hydrogen bonding.

### 3.4 Molecular docking of mRA probe binding site on Aβ protein

To understand how the mRA probe and Aβ_1 − 42_ bind, we conducted a molecular docking study. Investigating molecular interactions that stabilize protein-ligand complexes and locating ligand binding sites in proteins are made possible by molecular docking. Molecular docking simulations revealed that mRA exhibited significant binding affinities with Aβ_1 − 42_. mRA showed an affinity of −6.5 kcal/mol for Aβ_1 − 42_ with several close interactions. The docked conformations of mRA were ranked based on their binding affinities, with the top ones analyzed for their interaction profiles and binding stability. These findings suggest that mRA is an effective candidate for targeting Aβ_1 − 42_ in Alzheimer's disease. Notably, atomic-resolution structures of Aβ fibrils represent rigid, aggregated β-sheet assemblies with limited accessibility to conventional ligand binding pockets. Therefore, to explore the probable binding regions and affinity of mRA, we employed the monomeric Aβ structure, which provides better resolution of individual residue interactions. The docking results with monomeric Aβ_1 − 42_ should be interpreted as an initial model supporting the molecular recognition process that precedes aggregate formation and binding-induced fluorescence enhancement. Ligand efficiency is another crucial parameter that reflects the potency of a compound relative to its size and was also evaluated for mRA against Aβ_1 − 42_. The results demonstrated that mRA displayed notable ligand efficiency, underscoring its capability to effectively interact with Aβ_1 − 42_ with minimal molecular weight. This characteristic strengthens mRA's potential as an appreciable binding partner of Aβ_1 − 42_.

**Figure 6:**
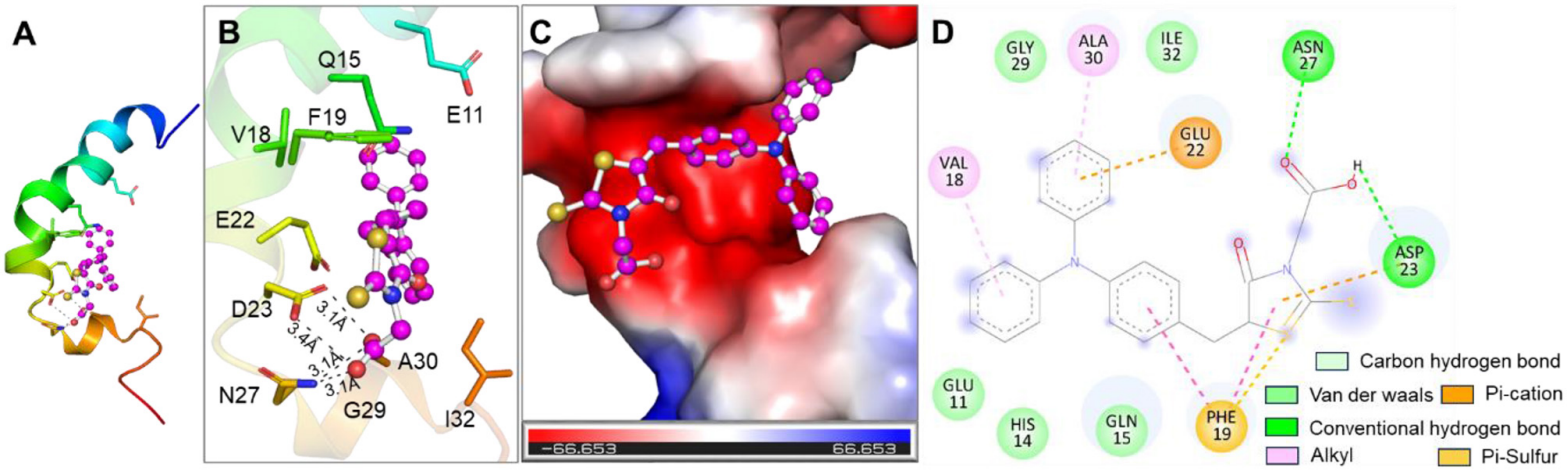
Molecular Interactions between mRA probe and Aβ. **(A)** Cartoon representation of the mRA probe binding site on Aβ. **(B)** Binding prototype of mRA probe with amino acid residues of Aβ. **(C)** Surface potential view of the Aβ binding pocket occupied by the mRA probe. **(D)** 2D structural representation of the nature of interaction of the mRA probe with the amino acid residues of Aβ.

Detailed analysis of the interactions between mRA and the binding sites of Aβ was performed and depicted in Figure 6. The docked poses of mRA revealed multiple significant interactions with key residues within the binding pockets of the target amyloid protein. These interactions included hydrogen bonds, hydrophobic contacts, and van der Waals forces, crucial for stabilizing the protein-ligand complexes. mRA engaged with several functionally essential residues within the Aβ_1 − 42_ binding pocket, forming distinct hydrogen bonds and hydrophobic interactions that contributed to its strong binding affinity. Aβ_1 − 42_ and mRA displayed close contact with critical residues, enhancing their binding stability (Figure 6A). A thorough examination of the docking poses showed that mRA occupied well-defined pockets in Aβ_1 − 42_, suggesting high specificity and affinity for these targets (Figure 6B). These findings are in good agreement with the fluorescence experiment results.

The binding site interactions were further analyzed and visualized using structural representations. A cartoon representation of Aβ demonstrated the positioning of mRA within its respective binding pockets, which revealed the molecular basis of the interaction. The surface potential views highlighted the regions of Aβ_1 − 42_ that mRA occupied, reinforcing the stability and compatibility of the docking poses (Figure 6C). The two-dimensional interaction diagrams of mRA probe with Aβ_1 − 42_ revealed hydrogen bonding and van der Waals interactions as the dominant forces stabilizing the complexes (Figure 6D). Docking studies revealed that probe mRA binds to Aβ through two conventional hydrogen bonds, Asp23 and Asn27, and a carbon-hydrogen bond at Ile32 stabilizes the probe mRA/Aβ complex. Gln15, Glu11, Val18, Phe19, Glu22, His14, Gly29, and Ala30 were also found to be involved in the interaction. mRA has significant binding affinity toward the Aβ at the N/C-terminal region, with a binding free energy of −6.5 kcal/mol for the mRA/Aβ complex formation. These interactions, coupled with favorable binding affinities and ligand efficiency, suggest that probe mRA holds significant potential as an effective binder of Aβ_1 − 42_. Table 3 lists the summary of fluorophores used for the sensitive detection of amyloid in Alzheimer's disease. To prevent photo-oxidation at 4°C, the mRA probe is kept in the dark and is incredibly stable. This study found good specificity and sensitivity in the quick detection of amyloid in bodily fluids, although various fluorescent probes have been reported.

**Table 3:** Summary of various types of fluorophores used for the sensitive detection of Aβ.

**Probe Name**	**Structure**	**Method used**	**Fold enhancement**	**Ref**
Pyridothiazole derivative	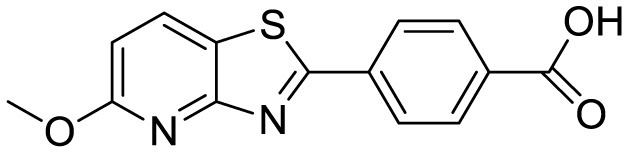	Cu(II) induced turn-on fluorescence	1μM	(Gour et al., 2018)
Hemicyanine-based benzothiazole-coumarin	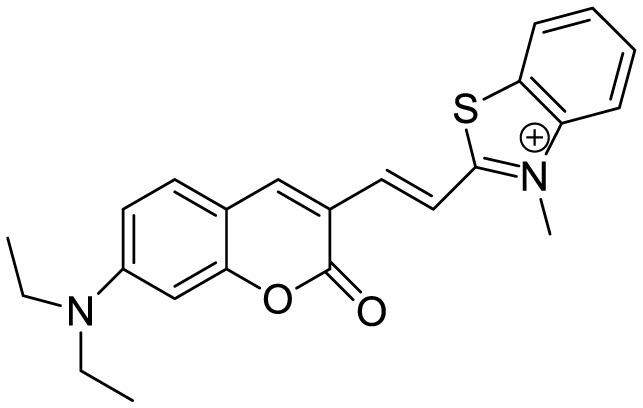	Aβ_42_ aggregates through switch-on, enhanced fluorescence	30	(Rajasekhar et al., 2016)
PTPA-QM	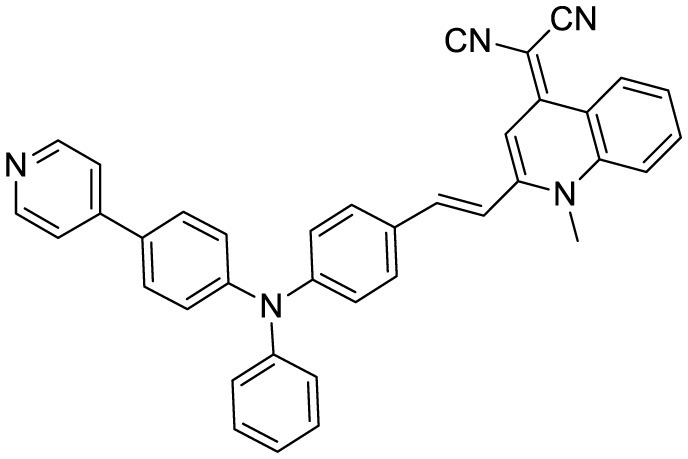	Aggregation-induced emission fluorescent probe,	22	(Fang et al., 2023)
N,N-dimethyl biannulated electron-donating groups: BZ-OB (benzofuran) and NAP-OB (naphthalene)	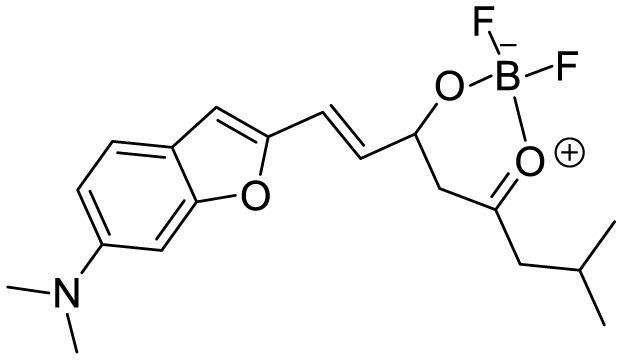 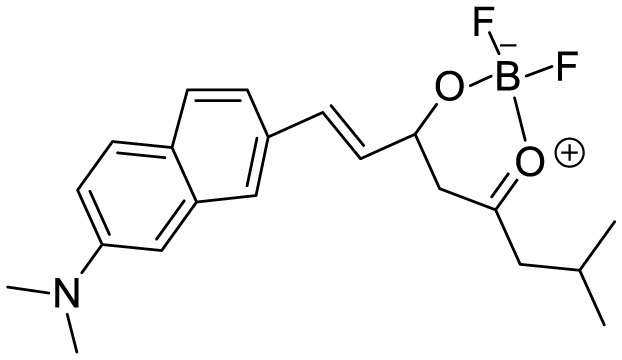	Polarity induced fluorescence/Fluorescence Imaging	20.5(BZ-OB) 69.4 (NAP-OB)	(An et al., 2022)
quinoline-based AIE probe(FB)	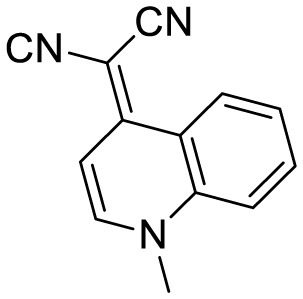	Aggregation-induced emission	20	(Wang et al., 2020)
2,1,3-benzothiadiazole (BTD)	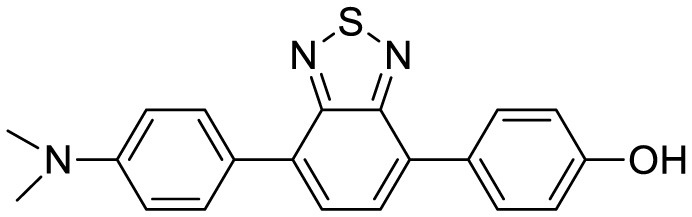	Two-photon fluorescence imaging	6	(Chen et al., 2024)
NPBZ	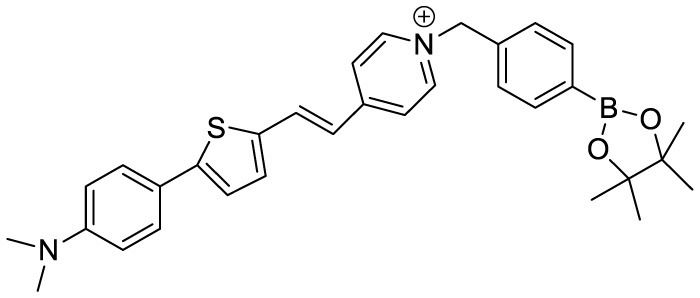	*in vivo* imaging	47	(Lv et al., 2025)
BPM1	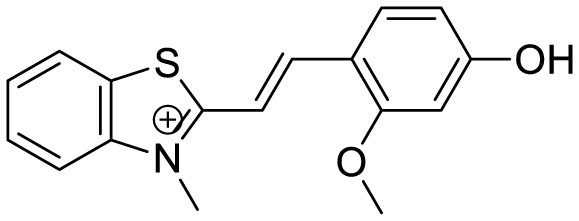	Aβ Aggregation-induced emission	10	(Zhao et al., 2023)
LDS722	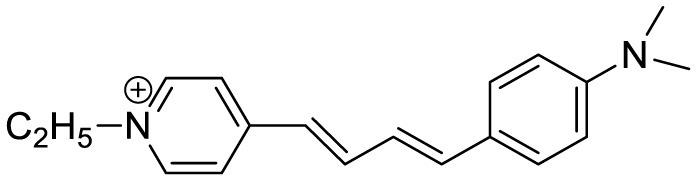	Fluorescence turn-on emission	200	(Warerkar et al., 2021)
1,7-bis[(2,5-dimethoxy-3- diethylamino)phenyl]-1,6-heptadiene−3,5-dione (curcumin-based NIR Dye)	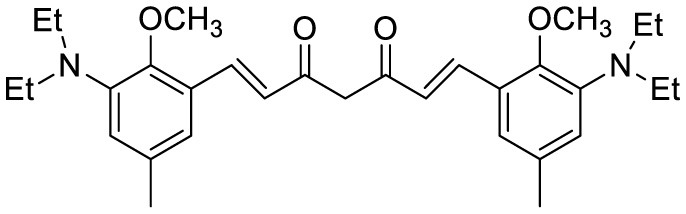	Fluorescence turn-on emission	19.5	(Si et al., 2019)
Thioflavin X	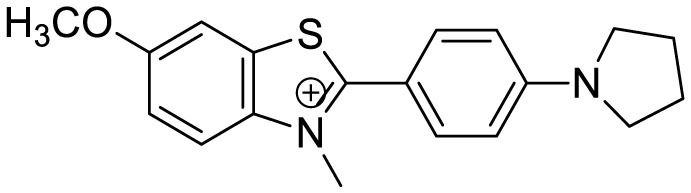	Aβ Aggregation-induced emission/ Fluorescence Imaging	7	(Needham et al., 2020)
Diethyl 6-(dimethylamino) naphthalene2,3-dicarboxylate (DMNDC)	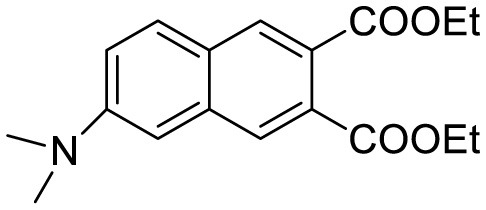	Aβ Aggregation-induced intramolecular charge transfer (ICT) emission	10	(Das et al., 2020)
(2-((1E,3E)-4-(4-(dimethylamino)phenyl)buta-1,3-dien-1-yl)- 1,1,3-trimethyl-1H-benzo[e]indol-3-ium iodide)	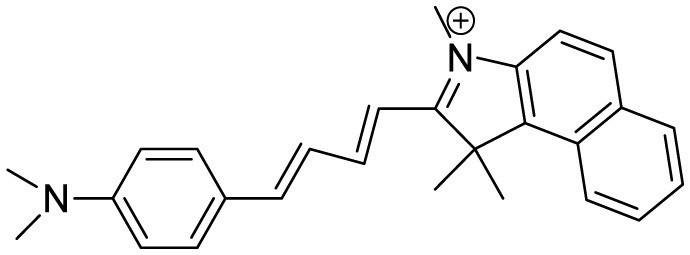	Aβ Aggregation-induced emission/ Fluorescence Imaging	–	(Lee et al., 2019)
THK-565	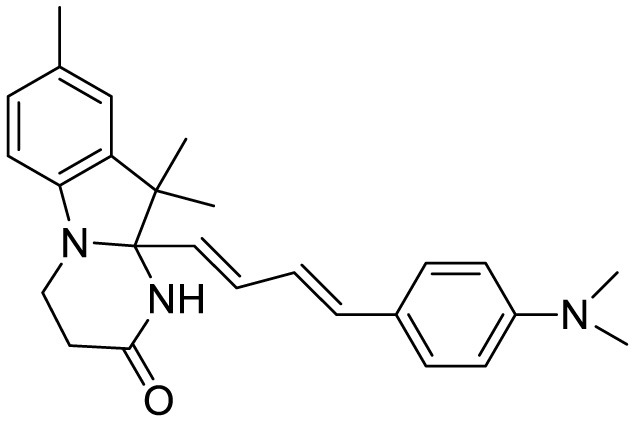	Fluorescence Imaging	16	(Naganuma et al., 2023)
Triphenylamine rhodamine-3-acetic (mRA)	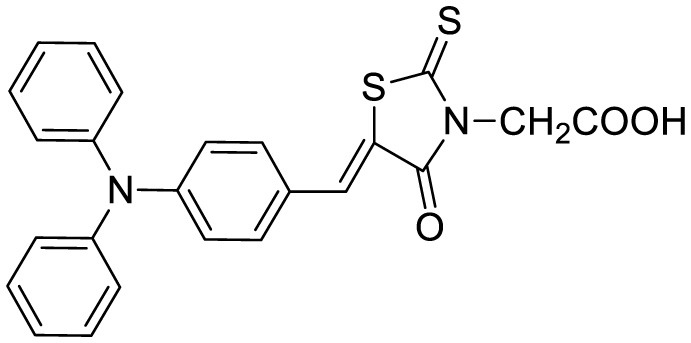	Fluorescence turn-on emission	24	This work

### 3.5 Specificity and cross-activity

**Figure 7:**
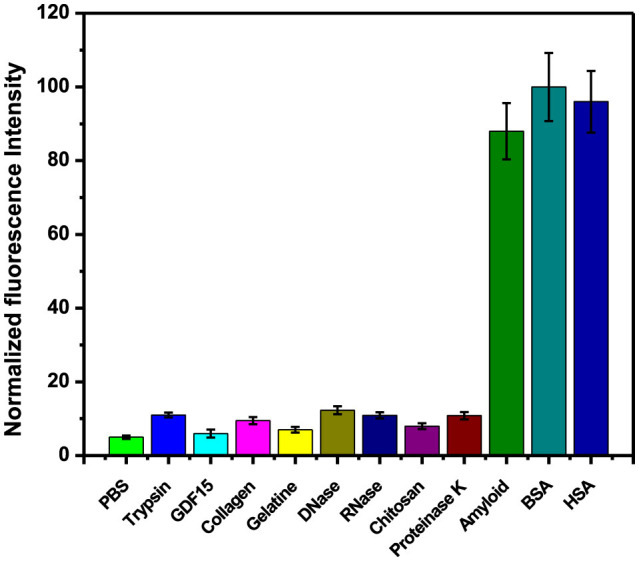
The cross-reactivity response of amyloid-specific mRA against other molecules such as trypsin, GDF15, collagen, gelatine, DNase, RNase, chitosan, proteinase K, BSA, and HAS.

The specific recognition of the Aβ protein in complex biological fluids by the molecular probe is a crucial factor for accurately detecting the target. As a consequence, we observed the fluorescence intensity of mRA treated with several other interfering molecular species, such as trypsin, GDF-15, collagen, gelatin, DNase, RNase, chitosan, bovine serum albumin (BSA), and human serum albumin (HSA), under the same conditions. As illustrated in Figure 7, HSA and BSA exhibited a significant increase in the fluorescence emission. Most of the biomolecules showed an insignificant influence on the fluorescence enhancement, except Aβ, as well as BSA and HSA. The mechanism of mRA fluorescence enhancement in HSA is due to the site-specific binding of mRA in binding site IIIA of the HSA protein. The mRA interaction sites in the binding site lock the intermolecular mobility of mRA, which triggers the fluorescence emission in the mRA/HSA complex (Chinnappan et al., 2025). The influence of the fluorescence signal by BSA is due to its sequence similarity with HSA, which would bind mRA in the same way. These results indicate that mRA is a potential fluorescent probe for the sensitive and specific detection of Aβ_1 − 42_ protein from biological fluids.

## 4 Conclusions

This study systematically characterized the interaction mechanism responsible for the mRA biosensor with Aβ_1 − 42_. mRA is proposed as a recognition molecule that shows outstanding advantages in Aβ binding and early detection. Binding of mRA to Aβ_1 − 42_ was investigated by the fluorescence spectroscopy results, which revealed strong interaction (*K*_a_ = 3.0 × 10^−6^ M^−1^) at 298 K. A change in fluorescence and a significant reduction in affinity on increasing temperature showed that mRA/Aβ complex formation results in static fluorescence enhancement. Additionally, we obtained a negative ΔG value from the Van't Hoff equation, which indicated that the reaction was spontaneous and thermodynamically beneficial. Data from the thermodynamics of mRA with Aβ showed that hydrogen bonds and van der Waals interactions are the main ways that mRA and Aβ_1 − 42_ protein interact. Aβ far-UV CD spectra went upward in the presence of mRA, suggesting the formation of the Aβ_1 − 42_/mRA complex. The results of thermostability and fluorescence binding demonstrate that mRA attaches to Aβ_1 − 42_ with a high binding energy and uses a variety of bonding forces to make several tight contacts with crucial residues.

In summary, this study demonstrates the binding mechanism of mRA to Aβ_1 − 42_. This is the first study to look at the mechanism of interaction between the therapeutically relevant mRA and the Aβ_1 − 42_ protein, which is important in AD. Understanding the mechanism of interaction between mRA and Aβ_1 − 42_ will aid in comprehending the forces that lead to this connection, and the particulars of these forces result in the construction of a stable complex. This study provides insight into the binding mechanism of mRA with Aβ_1 − 42_ and can be very helpful for the early detection and progression of amyloid accumulation in AD. Their biological significance and applications in targeting the Aβ_1 − 42_ to detect progression and accumulation in AD may become clearer with more research into the unique structural traits and functional consequences of mRA in clinical samples with cells of amyloid translation beginning. These findings have strengthened our confidence in the early diagnosis and treatment of AD in the analysis of Aβ_1 − 42_ aggregates, the development of new identification probes, and the quantification of Aβ_1 − 42_. This is a preliminary study of a specific fluorescent probe against Aβ. More detailed research is necessary to understand mRA and Aβ_1 − 42_ interactions for implementing mRA in AD-related clinical applications.

## Data Availability

The original contributions presented in the study are included in the article/Supplementary material, further inquiries can be directed to the corresponding author/s.
