# Supplementary material for: A novel fluorescent probe, triphenylamine rhodamine-3-acetic acid (mRA) for the detection of Amyloid-β aggregates in Alzheimer's disease
Source: Front Neurosci. 2025 Aug 21;19:1653063. doi: 10.3389/fnins.2025.1653063 (PMC12408501; doi:10.3389/fnins.2025.1653063)
Supplement: Supplementary file 1 [file Data_Sheet_1.docx]

**Supporting Information**

**A Novel Fluorescent Probe Triphenylamine Rhodamine-3-Acetic Acid (mRA) for the Detection of Amyloid-β Aggregates in Alzheimer’s Disease**

Raja Chinnappan^1,6*^, Mateen A. Khan^2,*^, Taj Mohammad^3^, Sarah Mohammed Allwaibh^2^, Shanmugam Easwaramoorthi^4^, Ahmed Yaqinuddin^1^, Sandhanasamy Devansan^5^, Tanveer Ahmad Mir^1,6^, and Md. Imtaiyaz Hassan^3^

^1^College of Medicine, Alfaisal University, Riyadh 11533, Saudi Arabia

^2^Department of Life Sciences, College of Science & General Studies, Alfaisal University, Riyadh 11533, Saudi Arabia.

^3^Center for Interdisciplinary Research in Basic Sciences, Jamia Millia Islamia, Jamia Nagar, New Delhi 110025, India.

^4^Inorganic and Physical Chemistry Lab, CSIR-Central Leather Research Institute, Sardar Patel Road, Adyar, Chennai 600020, India.

^5^Bioproducts Research Chair, Department of Zoology, College of Science, King Saud University, Riyadh, Saudi Arabia.

^6^Tissue/Organ Bioengineering & BioMEMS Laboratory, Organ Transplant Centre of Excellence (TR & I-Dept), King Faisal Specialist Hospital and Research Centre, Riyadh 11211, 11, Saudi Arabia.

*Corresponding authors: Email addresses: [matkhan@alfaisal.edu](mailto:matkhan@alfaisal.edu) (M.A. Khan) and [rchinnappan@alfaisal.edu](mailto:rchinnappan@alfaisal.edu) ( R.Chinnappan)

**Figure S1:** UV-visible absorption spectrum of mRA in water(Thamaraiselvi et al., 2019)

**Figure S2:** UV-visible absorption and fluorescence spectra of mRA in PBS and DMSO. The fluorescence spectra were measured by exciting the sample at 475 nm (Chinnappan et al., 2025).

**References:**

Chinnappan, R., Mir, T. A., Easwaramoorthi, S., Sunil, G., Feba, A., Kanagasabai, B., et al. (2025). Molecular engineering of a fluorescent probe for highly efficient detection of human serum albumin in biological fluid. *Sens. Int.* 6, 100304. doi: 10.1016/j.sintl.2024.100304

Thamaraiselvi, P., Duraipandy, N., Kiran, M. S., and Easwaramoorthi, S. (2019). Triarylamine rhodanine derivatives as red emissive sensor for discriminative detection of Ag+ and Hg2+ ions in buffer-free aqueous solutions. *ACS Sustain. Chem. Eng.* 7, 9865–9874.
